# Glyphosate Detection by Means of a Voltammetric Electronic Tongue and Discrimination of Potential Interferents

**DOI:** 10.3390/s121217553

**Published:** 2012-12-18

**Authors:** Román Bataller, Inmaculada Campos, Nicolas Laguarda-Miro, Miguel Alcañiz, Juan Soto, Ramón Martínez-Máñez, Luís Gil, Eduardo García-Breijo, Javier Ibáñez-Civera

**Affiliations:** 1 Centro de Reconocimiento Molecular y Desarrollo Tecnológico (IDM), Unidad Mixta Universidad Politécnica de Valencia–Universidad de Valencia de Valéncia, Camino de Vera s/n, E-46022 Valencia, Spain; E-Mail: robapra@upvnet.upv.es; 2 Departamento de Química, Universidad Politécnica de Valencia, Camino de Vera s/n, E-46022 Valencia, Spain; E-Mails: incasan2@upvnet.upv.es (I.C.); jsotoca@upv.es (J.S.); rmaez@upv.es (R.M.M.); 3 CIBER de Bioingeniería, Biomateriales y Nano medicina (CIBER-BBN), Bellaterra, E-08193 Barcelona, Spain; 4 Departamento de Ingeniería Química y Nuclear, Universidad Politécnica de Valencia, Camino de Vera, s/n, E-46022 Valencia, Spain; E-Mail: milami@iqn.upv.es; 5 Departamento de Ingeniería Electrónica. Universidad Politécnica de Valencia. Camino de Vera, s/n, E-46022 Valencia, Spain; E-Mails: mialcan@upvnet.upv.es (M.A.); lgil@eln.upv.es (L.G.); egarciab@eln.upv.es (E.G.B.); jibanyez@eln.upv.es (J.I.C.)

**Keywords:** glyphosate, electronic tongue, rotating disk electrodes, PLS

## Abstract

A new electronic tongue to monitor the presence of glyphosate (a non-selective systemic herbicide) has been developed. It is based on pulse voltammetry and consists in an array of three working electrodes (Pt, Co and Cu) encapsulated on a methacrylate cylinder. The electrochemical response of the sensing array was characteristic of the presence of glyphosate in buffered water (phosphate buffer 0.1 mol·dm^−3^, pH 6.7). Rotating disc electrode (RDE) studies were carried out with Pt, Co and Cu electrodes in water at room temperature and at pH 6.7 using 0.1 mol·dm^−3^ of phosphate as a buffer. In the presence of glyphosate, the corrosion current of the Cu and Co electrodes increased significantly, probably due to the formation of Cu^2+^ or Co^2+^ complexes. The pulse array waveform for the voltammetric tongue was designed by taking into account some of the redox processes observed in the electrochemical studies. The PCA statistical analysis required four dimensions to explain 95% of variance. Moreover, a two-dimensional representation of the two principal components differentiated the water mixtures containing glyphosate. Furthermore, the PLS statistical analyses allowed the creation of a model to correlate the electrochemical response of the electrodes with glyphosate concentrations, even in the presence of potential interferents such as humic acids and Ca^2+^. The system offers a PLS prediction model for glyphosate detection with values of 098, −2.3 × 10^−5^ and 0.94 for the slope, the intercept and the regression coefficient, respectively, which is in agreement with the good fit between the predicted and measured concentrations. The results suggest the feasibility of this system to help develop electronic tongues for glyphosate detection.

## 1. Introduction

**Figure 1.**
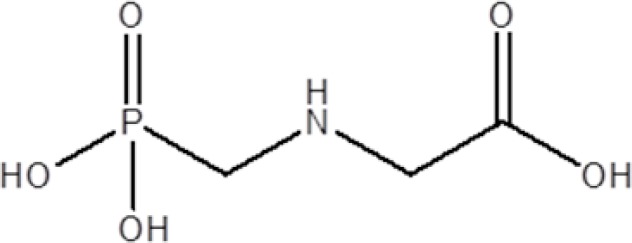
Chemical structure of glyphosate.

Glyphosate (see Figure 1) is a non-selective broad-spectrum systemic herbicide of widespread use in agriculture [1]. It operates by inhibiting the enzyme 5-enolpyruvylshikimate-3-phosphate synthase, also known as EPSP, which performs an essential function for the synthesis of amino acids vital to plant life, such as tryptophan and tyrosine [1].

The use of this herbicide is strictly controlled in Spain [2] and in the European Union [3], but it is still used without much control over vast rural areas worldwide. In these regions, this herbicide is applied by spraying it with light aircrafts; hence, the application area selectivity is poor, as the product not only settles on the crop to be treated, but also precipitates on other non-desired areas, affecting water [4,5], soil, [6], wildlife, as well as humans directly and indirectly [7].

Glyphosate often presents an analytical challenge because of its relatively high solubility in water, insolubility in organic solvents, high polarity and low volatility. In the literature, several works can be found that develop methodologies for glyphosate detection. Most of these studies are based on chromatographic techniques using either gas [8,9] or liquid chromatography (HPLC) [10–13]. In the case of the studies using HPLC chromatography, the reached limits of detection were in the range of μg/mL when the sample was conditioned by pre- or post-column derivatization protocols and coupled to mass analysis (HPLC/MS/MS). Another reported approach found in the literature to detect glyphosate was its derivatization with a chromophore or fluorophore and the use of techniques such as UV [14], fluorescence or diffuse reflectance spectroscopy [15]. Other methodologies without derivation such as capillary electrophoresis [16], electrochemiluminescence [17], conductivity detection and inductively coupled plasma/mass spectrometry (ICP/MS) [18] have been reported for the detection of glyphosate. Besides, biosensors using colorimetric or electrochemical detection protocols have also been reported for glyphosate sensing. Biosensors present advantages such as having high sensitivity, specificity, easy operation and relatively low cost. However as drawbacks biosensors can be difficult to prepare and maintain, as usually antibodies require controlled conditions for optimal operation [19,20]. Other techniques using double layer hydroxide electrochemical sensors have also been reported in the literature [21,22]. Nevertheless, these procedures are generally slow, expensive and have to be performed in a laboratory (are not suitable for *on-site* or *in-situ* monitoring). In this context advances in the design and development of rapid, sensitive and easy-to-use analytical procedures for glyphosate detection are of importance [17]. In fact, some interesting advances have already been reported, such as those by Aquino *et al*. [23] based on electrochemical procedures using silver, platinum [22] or copper [23] electrodes and reactive enzymes [24], which could be the basis for future development of equipment for the easy detection of this herbicide. Although the results of these works are encouraging, they do not examine the possible effects of matrix composition on the detection and quantification of glyphosate. In fact, there are several substances present in water that may interact while performing different glyphosate detection methods. In the specific case of electrochemical methods, humic substances may act as interferents. These compounds are the dominating forms of organic carbon in soil and aqueous ecosystems [25], they are often present in the environment as non-easily identifiable structures [26], and are important in the task of accumulating, transforming and eliminating colloids, nutrients and pollutants [27] from the environment [28,29]. There have been several studies about humic substances in the environment, soil and water, and most indicate that their behaviour is quite difficult to predict [28] and that it depends on several variables, such as the presence/absence of other compounds or chemicals; e.g., Ca^+2^, a cation present in natural and run-off waters. Moreover, this cation has the ability to form stable Ca-glyphosate complexes [27,29].

Electronic tongues have recently emerged as a potential simple, low-cost tool for analysis purposes. Electronic tongues use semi-specific sensors that produce a signal pattern when subjected to a sample, which can be related to either a specific compound or a quality aspect. [30] Such characteristics make these systems unique in the analytical systems field; thus, their application to various fields of interest is continuously growing [31,32]. Following our interest in the development of new electrodes and electronic tongue devices [33–40] for their application in a wide range of problems, we report herein the design of a voltammetric electronic tongue which consists in three metallic electrodes (*i.e.*, Co, Cu and Pt) and their use in detecting glyphosate in aqueous samples. Multivariate analyses, including principal component analysis (PCA) and partial least square (PLS) techniques have been applied to build suitable management and prediction models for the determination of glyphosate concentrations. Besides, it has also been studied the effect of calcium and humic acids in glyphosate detection.

## 2. Experimental

### 2.1. Sample Preparation

Two types of analysis have been carried out: discrimination of samples and prediction of concentration levels. Discrimination studies were carried out using two sets of samples. The first set contained four different samples consisting of: (i) phosphate buffer (0.1 mol·dm^−3^ at pH 6.7); (ii) phosphate buffer and 20 mg·dm^−3^ of a domestic plant fertilizer containing humic acids (manufacturer Massó Garden, Valencia, Spain; composition: 4.5% w/w total N, 1.75% w/w organic N, 2.75% w/w ammoniacal N, 3% w/w K_2_O, 22% w/w organic C); (iii) phosphate buffer and 40 mg·dm^−3^ of CaCl_2_ (with 96% of purity from Aldrich, Valencia, Spain); and (iv) phosphate buffer, 20 mg·dm^−3^ of humic acids and 40 mg·dm^−3^ of CaCl_2_. The second set of samples was prepared similarly, but also contained glyphosate at a concentration of 5 × 10^−4^ mol·dm^−3^.

In the first part of the prediction of glyphosate concentration levels, 11 glyphosate samples were used, in a concentration range of between 0 and 5 × 10^−3^ mol·dm^−3^ (2.5 × 10^−5^, 5 × 10^−5^, 6.25 × 10^−5^, 1.25 × 10^−4^, 2.5 × 10^−4^, 5 × 10^−4^, 1.25 × 10^−3^, 2.5 × 10^−3^, 5 × 10^−3^). These solutions were buffered with phosphate at a concentration of 0.1 mol·dm^−3^ and at pH 6.7. Each sample was measured three times. PLS analyses were performed on the 33 measured samples (11 samples × three repetitions). The analysis was also made for each measuring strategy (rotating or static electrodes). A set of 21 samples (three trials of seven samples) was used as the training set, whereas the remaining 12 samples were used to validate the model. Calibration and validation samples were selected randomly. Each sample was measured five times and the mean was applied in a pre-processing step.

In a second part, studies of the potential interferents were done and the prediction of the glyphosate concentration levels was carried out in the presence of humic acids and calcium. Samples preparation was performed using the MODDE 8.0 experimental design program (Umetrics, Umea, Sweden). With this program, a system of three compounds (glyphosate, humic acids and Ca^2+^) was established at three concentration levels. The final set included 19 samples prepared by addition of three selected compounds (glyphosate, humic acids and calcium chloride) into water (phosphate buffer 0.1 mol·dm^−3^, pH 6.7). The lower level was 1 × 10^−4^ mol·dm^−3^ for glyphosate, and 0 and 10 ppm for humic acids and calcium, respectively. The medium level contained 5 × 10^−4^ mol·dm^−3^ for glyphosate, 10 ppm of humic acids and 25 ppm of calcium. The higher level consisted in 1 × 10^−3^ mol·dm^−3^ for glyphosate, and in 20 and 40 ppm for humic acids and calcium, respectively (see Table 1).

For this study, the 19 solutions were measured three times, thus obtaining a total of 57 samples, 39 (three trials of 13 samples) of which were used for training 18 samples for the validation experiments.

### 2.2. Electrochemical Studies

Electrochemical experiments were performed using an Autolab PGSTAT100 instrument (Echo Chemie, Utrecht, The Netherlands). Electrochemical characterization was carried out using aqueous solutions of 2.5 mmol·dm^−3^ of glyphosate. The solutions at pH 6.7 were buffered with phosphate buffer at a concentration of 0.1 mol dm^−3^. All the measurements were taken at room temperature (25 ± 2 °C) using Pt, Cu and Co as the working electrodes. A platinum electrode and a saturated calomel electrode were used as the counter and reference electrode, respectively. The rotating disk electrodes (RDE) studies were performed at a scan rate of 10 mVs^−1^ and at a rotation velocity of 1,500 rpm.

### 2.3. The Electronic Tongue

#### 2.3.1. The Electronic System

The electronic system used in the electronic tongue was developed in the IDM Research Institute at the Polytechnic University of Valencia (E. Spain). The system consisted in a software application, which runs on a PC, and electronic equipment. The system includes a potentiostat that applies the voltage to the electrochemical cell, and measures the voltage and the current at the working electrodes. The potentiostat permits measurements with up to 8 multiplexed working electrodes. The electronic equipment includes a 16-bit microcontroller (PIC24FJ256), a 12-bit Digital-to-Analog converter (DAC), two 12-bit Analog-to-Digital converters (ADC) and a potentiostat that incorporates a current measurement circuit, a working electrode multiplexing block and a stabilization circuit. Some analog signal conditioning circuits are used to adapt the signals that connect the potentiostat to the DAC and the ADC. The microcontroller receives the data sent by the PC. When all the data corresponding to a test are received, the microcontroller configures the current scale and the stabilization level of the potentiostat, and then selects the desired working electrode. Then it outputs the value corresponding to the temporal evolution of the signal to the DAC at a rate that fulfils the signal timing requirements. In the same loop, the program of the microcontroler samples the signals corresponding to the voltage and the current flowing at the selected working electrode. The collected data are sent to the PC where they are processed and stored.

#### 2.3.2. E-Tongue Preparation

The electronic tongue device consists in an array of three working electrodes (Pt, Cu and Co) with 99.9% purity and 1 mm diameter from Aldrich, which were housed inside two homemade methacrylate cylinders, used as e-body of the electronic tongue system. The different wire electrodes were fixed inside the cylinder using an epoxy RS 199-1468 polymer. Pulses were generated and current data were recorded using the above-described electronic equipment. One advantage of utilizing electronic tongues based on metallic electrodes is that it is quite simple to prevent fouling problems by simply polishing the electrodes. A commercial platinum electrode and a saturated calomel electrode (SCE) were used as the counter and reference electrode, respectively. In other to eliminate the instrumental drift that characterize the electronic tongues, working electrode surfaces were prepared by mechanical polishing with emery paper and were rinsed with distilled water before their use. Then they were polished on a felt pad with 0.05 μm alumina polish from BAS, washed with distilled water and polished again on a nylon pad with 15, 3 and 1 μm diamond polishes to produce a smooth, mirror-like electrode surface. Later while taking a series of measurements, only simple diamond polishing was done.

#### 2.3.3. Measurement Procedure

Samples were measured using the equipment developed in the IDM and the aforementioned electrodes. The applied pulse sequence was formed by nine pulses: 0, −200, 0, 600, −500, −200, 0, 200, 0 (mV). Each pulse was applied for 40 ms. A total of 414 currents (23 points per pulse × 9 pulses × 2 electrodes) were recorded for each sample. The time required to complete the measure with five repetitions was only two seconds.

#### 2.3.4. Data Management; Multivariate Analysis

A multivariate data analysis (MVDA) is used to process the raw data obtained from the instruments. A principal Component Analysis (PCA) is an example of such an MVDA, which explains the variance in the experimental data [41]. The PCA produces a score plot that visualizes the differences between observations or experiments. This can be used to classify or group observations. The first principal component (PC1) is the dimension along which observations are maximally separated or spread out. The second principal component (PC2) is the linear combination with maximal variance, which is in an orthogonal direction to PC1, and so on [42].

For the sensory data, means were calculated and statistically tested using an analysis of variance to determine if there was a statistical difference at P < 0.05, and Duncan’s multiple range tests were used to identify the statistical separation among the means [43]. For the instrumental data, the data matrix is where the number of objects is decided by the number of experiments, and the variables are the current responses created by the applied potential. [44] This data matrix is processed with a PCA. They are standardized since standardization gives all the variables the same variance, and all the variables have the same influence on the estimation of the components.

For the quantitative studies, the multivariate analysis used to process all the data collected from the experiment was performed by following the Partial Least Square (PLS) method. The aim of PLS is to predict Y from X by the simultaneous decomposition of those matrices or vectors in a group of components (latent variables) to explain the covariance of X and Y as much as possible [45]. The prediction models for glyphosate in both the matrix samples were built by using the data collected from the electronic tongue in the calibration set. Prior to building the model, cross-validation was used to evaluate the adequacy of the experimental data and for selecting the quantity of latent variables. The model obtained for these parameters was then applied to the set of validation samples. Model evaluation was carried out by comparing real *versus* predicted concentrations using the correlation coefficient (r2), p1, p2 (from y = p1x + p2 in the simple lineal model) and the root mean square error of prediction (RMSEP). All the studies were performed with the Solo software (version 6.5, Eigenvector Research, Inc., Wenatchee, Washington, DC, USA).

## 3. Results and Discussion

### 3.1. RDE Voltammetry Studies

Prior to the electronic tongue experiments, an electrochemical study was carried out to determine the electrochemical behaviour of glyphosate using the metallic electrodes Co, Cu and Pt. The study was conducted in water at room temperature and at pH 6.7 using 0.1 mol·dm^−3^ of phosphate as the buffer. As is well-known, the electrochemical response of a given compound depends on the intrinsic chemical nature of both the electrode and the redox behaviour of the product itself. In addition, each electrode’s transient response depends on the diffusion coefficients of the oxidized or reduced species, and also on the possible presence of specific chemical or electrochemical reactions between the electrode and the redox-active species. In fact, it has been reported that glyphosate forms complexes with Cu(II) and Co(II) metal cations. Indeed, as Figure 1 shows, the chemical structure of glyphosate contains three potentially coordinating groups: amine, carboxylate and phosphonate moieties; therefore, the herbicide is able to form chelating structures with different metal ions and surfaces [46,47].

**Figure 2.**
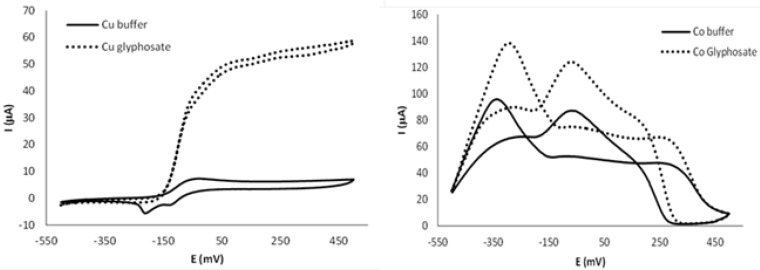
Copper and cobalt rotating disk electrode voltammograms of the solvent at pH 6.7, buffered with 0.1 M Na_2_PO_4_ (solid line) and 2.5 mM of glyphosate (discontinuous line) measured at 10 mV·s^−1^ and 1,500 rpm.

In the electrochemical experiments, the rotating disk voltammogram of the copper electrode showed an irreversible oxidation process at −80 mV corresponding to Cu^2+^ formation (see Figure 2). Two peaks appeared when potential was reversed; the first one corresponded to the reduction of Cu^2+^ to Cu^+^, and the second to reduction to Cu^0^. In the presence of glyphosate, the corrosion current of copper increased significantly, probably due to the Cu^2+^ complexation by glyphosate forming soluble species to result in the electrochemical dissolution of copper. The rotating disk voltammogram of the cobalt electrode in the absence of glyphosate showed how oxidation of the metal started at −500 mV. In this case however, a complex RDE curve was obtained at higher potentials, suggesting intricate electrochemical behaviour with the formation of several species at the electrode, most likely involving different hydroxide and oxide cobalt species. In the presence of glyphosate, the corrosion current increased, which was in agreement with the Co^2+^-glyphosate complexes formation [46]. When using a rotating disk of platinum electrode, glyphosate did not display a well-defined redox process in water (electrochemical window in the −0.8 to +1.0 V range). Nevertheless, this electrode was used in the electronic tongue to obtain information about non-Faradic processes.

**Figure 3.**
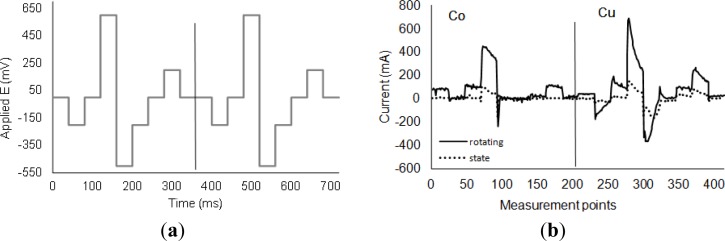
(**a**) Applied pulse sequence and (**b**) obtained response from glyphosate aqueous solutions (C = 5 × 10^−3^ mol·dm^−3^) using the Co and Cu electrodes (dashed line) and when the electrodes were rotating (continuous line) after subtracting the solvent signal.

The wave form for the electronic tongue (see Figure 3(a)) was designed by taking into account some of the redox processes observed in the electrochemical studies. Pulses 2 and 6 corresponded to the irreversible reduction peak of Cu^+^ to Cu^0^. Peaks at 600 mV (pulse 4) and at −500 mV (pulse 5) corresponded to the processes Cu^0^ to Cu^+2^ and the corresponding reduction Cu^2+^ to Cu^0^, respectively. At 200 mV, a cross appears in the baseline in the cobalt RDE (pulse 8). Moreover, pulses 1, 3, 7 and 9 were set to 0 mV, which corresponded to the maximum difference between the baseline and the glyphosate response in the Cu voltammogram. Figure 3(b) shows a typical response of both electrodes (Co and Cu) when applying the pulse sequence after subtracting the baseline. The figure shows two different responses corresponding to studies using static electrodes and rotating electrodes (1,500 rpm). The difference in the current signal between the rotating and static conditions is about 300 μA, indicating a greater response in the presence of glyphosate when rotating conditions are used.

### 3.2. PCA Analysis

The response of the voltammetric electronic tongue was studied in water (phosphate buffer, 0.1 mol·dm^−3^, pH 6.7) using different samples. Studies were also conducted in the presence of potential interferents, such as humic acids and Ca^2+^. Two sets of samples were prepared; the first consisted in four samples, *i.e.*, (i) phosphate buffer (0.1 mol·dm^−3^ at pH 6.7); (ii) phosphate buffer and 20 mg·dm^−3^ of humic acids; (iii) phosphate buffer and 40 mg·dm^−3^ of CaCl_2_; and (iv) phosphate buffer, 20 mg·dm^−3^ of humic acids and 40 mg·dm^−3^ of CaCl_2_; the second set of samples comprising the same solutions, but they also contained glyphosate at a concentration of 5 × 10^−4^ mol·dm^−3^. Each sample was measured three times. Moreover, two different electrochemical measurements were taken with the static and rotating (1,500 rpm) electrodes. In order to show the differential response of the tongue in the presence or absence of glyphosate, the electrochemical data was combined and studied using PCA analysis.

**Figure 4.**
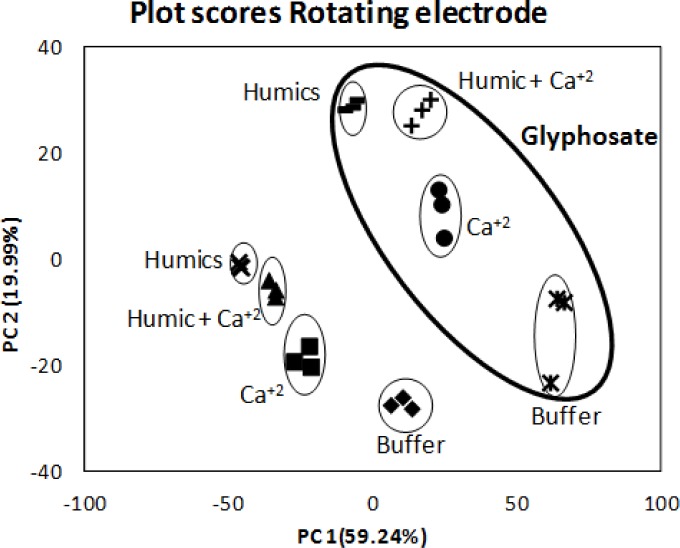
Principal component analysis score plot for the rotating electrodes on different aqueous samples.

The PCA score plot obtained for the rotating electrodes for the complete set of samples is provided in Figure 4. The PC1 contained only 52.87% and 59.24% of variance, and the first two components represented 79.12 and 79.23% of the total variance of the data when using the static (not shown) and rotating electrodes, respectively, whereas four PCs were needed to account for 95% of variance. Nevertheless, two dimensions (PC1 and PC2) in the PCA plots were enough to differentiate samples. In fact, the electronic tongue was able to discriminate the presence of glyphosate and those samples containing this herbicide appeared well separated in both assays using either the rotating (see Figure 4) or the static (not shown) electrodes. This is a promising result as it suggests that the differential response in the voltammetric technique using a set of electrodes might be a suitable method to detect glyphosate in aqueous samples.

### 3.3. PLS Analysis

The PCA study has shown that the data from the electronic tongue clustered according to glyphosate presence help obtain a good classification model, even in the presence of potential interferents such as humic acids and Ca^2+^. In this section, we were also interested in analyzing whether the data taken from the electronic tongue could be used to predict glyphosate concentration levels. In order to achieve this goal, the Partial Least Square (PLS) regression technique was used. In a pre-processing step, the baseline signal was subtracted from each sample signal. For this study, there were five latent variables, which were determined by cross-validation studies using one third of the whole data set used to validate the model. Eleven solutions of glyphosate within a concentration range of 0 and 5 × 10^−3^ mol·dm^−3^ were used. These solutions were buffered with phosphate buffer (pH 6.7). Each solution was measured three times. PLS analyses were performed with 33 samples (11 solutions × 3 repetitions) for each set of measurements using the rotating or static electrodes. In both cases, 21 samples (three trials of seven samples) were used as the training set, whereas the remaining 12 samples were used to validate calibration. The PLS model consists of a set of regression coefficients (βi) and residual value (E). This allows to obtain the value of glyphosate concentration from the measurement data. If we consider that x_1_, x_2_, …, x_n_ are the values corresponding to the temporal evolution of the current signal at the working electrodes for a certain sample then the value of glyphosate concentration for this sample can be calculated using Equation (1):

(1) $$C=E+\sum_{i=1}^{n}\beta_{i}\cdot x_{i}$$

Figure 3 shows the results of applying the PLS application to the calibration and validation sets when using the rotating or static electrodes. Additionally, Table 2 displays the adjusting parameters (r^2^, p1, p2 and RMSEP) from the PLS prediction model applied to the validation set for each created model.

**Figure 5.**
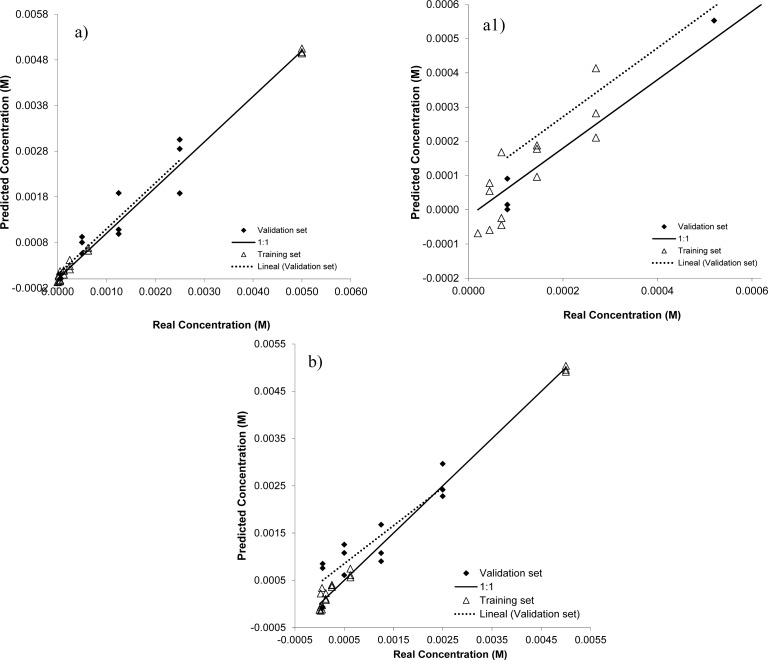
The results of the PLS prediction of glyphosate in phosphate buffer using (**a**) rotating electrodes, (**a1**) an extension of graph (a) and (**b**) PLS prediction of glyphosate using static electrodes.

Figure 5 allows a simple visual inspection of the accuracy and precision created in the prediction model applied to the validation set of glyphosate. Additionally, a more rigorous analysis was achieved by linearly fitting experimental points. We used a simple linear model (*i.e.*, y = p1x + p2) to adjust the predicted *versus* real concentration, and we obtained the parameters p1 (slope of the fitting line), p2 (intercept with the y axis) and RMSEP (root mean square of prediction). Parameters p1 and p2 related with accuracy in prediction, whereas RMSEP deals with the precision of the model. Thus, the PLS model is much better as the p1 value approached 1, while the RMSEP values came close to 0. Figure 5(A,B) depicts that the correlation obtained for the PLS model was better when using the rotating working electrodes (see also Table 2).

This observation could be confirmed if we compared the r^2^ values. For the models created with the rotating electrodes, r^2^ was 0.876, whereas r^2^ was 0.809 for the models created with the static electrodes. These results are in agreement with our first observation (see above) where we considered that an increase in the current signal when using the rotating electrodes could improve the accuracy and precision of the created system.

Encouraged by these results obtained while predicting the concentration levels of glyphosate in phosphate buffer, further experiments were carried out using a new set of samples containing humic acids and Ca^+2^ for the purpose of evaluating a possible matrix effect and for detecting glyphosate in the presence of these potential interferents. By bearing this aim in mind, samples selection was performed using the MODDE 8.0 experimental design programme (Umetrics). With this programme, a system of three compounds was established at three levels. The final set included 19 samples, prepared by the addition of different glyphosate, humic acids and calcium chloride concentrations in water with phosphate buffer at 0.1 mol·dm^−3^, pH 6.7 (see the Experimental Section for details). It has been shown above that the models created under rotating conditions provide better results. For this reason, only the studies using the rotating Pt, Co and Cu electrodes were carried out in this part of our work. Nineteen solutions were prepared and each solution was measured three times. From these solutions, 38 samples were used as the training set, whereas the remaining 19 samples were used to validate calibration. In the cross-validation studies, four latent variables were used. In this case, the glyphosate concentration was calculated as: $C=E+\sum_{i=1}^{621}\beta_{i}\cdot x_{i}$.

**Figure 6.**
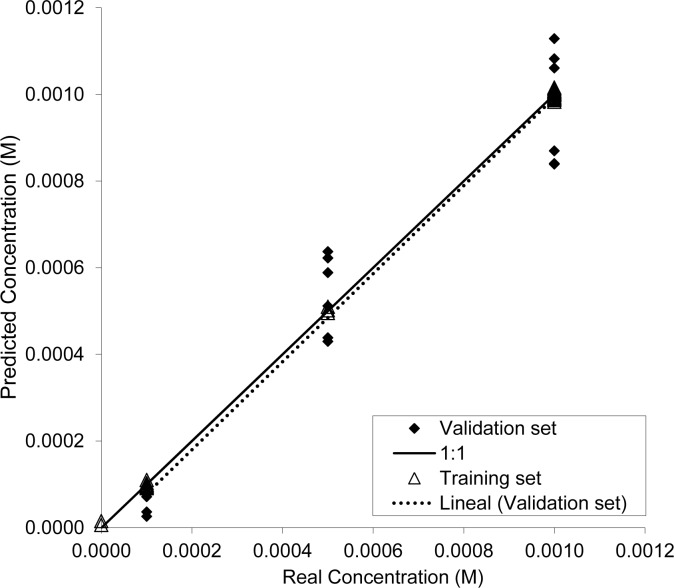
The results of the PLS prediction of glyphosate in phosphate buffer using the electronic tongue and rotating disk electrodes.

Table 3 shows the adjusting parameters from the PLS models created, whereas Figure 6 illustrates the PLS model obtained for glyphosate when using the electronic tongue. As seen in Table 3, adjusting parameters r^2^ and p1 came closer to 1 for glyphosate, demonstrating a good correlation between the electrochemical response of the electrodes and the different amounts of glyphosate in the water samples. Moreover, the attempts made to predict the concentrations of humic acids or Ca^2+^ were unsuccessful and poor r^2^ and values p1 were obtained in both cases (see Table 2). This absence of interference in the presence of these species may relate with a larger complexation constant between glyphosate and copper than with the complexation constants between humic acids and copper, and between glyphosate and calcium cations. These results indicate that the electronic tongue is quite capable of detecting this analyte, even in the presence of different concentrations of the potential interferents, such as humic acids and Ca^2+^.

## 4. Conclusions

A new voltammetric electronic tongue based on three metallic working electrodes (Pt, Co and Cu) encapsulated on a methacrylate cylinder for monitoring glyphosate has been developed. A suitable waveform for the voltammetric tongue was designed by taking into account some of the redox processes observed in the electrochemical studies. This electronic tongue is capable of distinguishing the presence of glyphosate with different buffered water samples following a recognition pattern based on the metallic electrodes’ cross-sensitivity. The PCA statistical analysis of the results confirmed the system’s ability to not only classify samples according to the presence of glyphosate, but to obtain samples clustering in a two-dimensional diagram without errors or misclassifications. The differential electrochemical response was also employed to create a PLS model, which showed a good quality fit between the predicted and observed values, as well as a regression coefficient of 0.94, even in the presence of interfering species such as humic acids and Ca^2+^. The results suggest the feasibility of this or similar voltammetric tongues to design systems that are able to detect moderate concentrations of this herbicide in aqueous environments.

## Figures and Tables

**Table 1.** Concentration of samples prepared by addition of three selected compounds.

**Sample**	**Glyphosate (mol·dm^−3^) × 10^−4^**	**Humic acids (mol·dm^−3^) × 10^−4^**	**Ca^2+^ (mol·dm^−3^) × 10^−4^**
1	10	10	1
2	1	10	10
3	5	5	5
4	10	1	5
5	1	10	1
6	10	5	10
7	10	10	10
8	10	10	10
9	10	10	10
10	1	1	1
11	1	1	10
12	10	1	10
13	10	10	10
14	1	1	5
15	5	1	10
16	5	1	1
17	10	1	1
18	10	1	10
19	0	0	0

**Table 2.** The adjusting parameters (r2, p1, p2 and RMSEP) from the PLS prediction models for the data from the validation set.

	**r^2^**	***p1***	***p2***	**RMSEP**
Rotating electrodes	0.876	1.01	9.1 × 10^−5^	3.6 × 10^−4^
Static electrodes	0.809	0.805	4.5 × 10^−4^	4.7 × 10^−4^

**Table 3.** The adjusting parameters (r^2^, p1, p2 and RMSEP) from the PLS prediction models for the data from the validation set.

	**r^2^**	***p1***	***p2***	**RMSEP**
Glyphosate	0.94	0.98	−2.3 × 10^−5^	9.5 × 10^−5^
Humic acids	0.11	0.16	6.9	9.10
Ca^+2^	0.13	0.47	−0.93	22.18
